# Supplementary material for: Enhanced Senescence Process is the Major Factor Stopping Spike Differentiation of Wheat Mutant ptsd1
Source: Int J Mol Sci. 2019 Sep 19;20(18):4642. doi: 10.3390/ijms20184642 (PMC6770497; doi:10.3390/ijms20184642)
Supplement: Supplementary file 1 [file ijms-20-04642-s001.zip › Figures S1-S13.doc]

**Figure S1** Illustration of the proposed scales of wheat spike differentiation of WT (**A**–**F**) and mutant *ptsd1* (**G**–**L**). **(A,G)** Early double ridge stage. **(B,H)** Double ridge stage. **(C,I)** Late double ridge stage. **(D,J)** Glume primordium visible. **(E,K)** Terminal spikelet stage. **(F,L)** Initiation of second awn. The red parentheses in pictures (**I**–**L)** indicate the upper undifferentiated spikelets. Scale bar = 500 μm.

**Figure S2** Spike histological sections of WT and mutant *ptsd1.* Spike histological sections of WT **(A,C)** and mutant *ptsd1* **(B,D)** [stained with a mixture of safranin and fast green](http://xueshu.baidu.com/s?wd=paperuri:(7dc006dc62d5d7068899193e126c8c79)&filter=sc_long_sign&sc_ks_para=q=Differential Staining with a Mixture of Safranin and Fast Green FCF&sc_us=15607579984830896637&tn=SE_baiduxueshu_c1gjeupa&ie=utf-8). **(A,B)** Late double ridge stage. **(C,D)** Initiation of second awn. im, inflorescence meristem; spp, spikelet primordium; spd, spikelet meristematic dome; glp, glume primordium; lemp, lemma primordium; flp, floret primordium; stp, stamen primordium; pip, pistil primordium. Red arrowheads indicate the undifferentiated spikelets. Scale bar = 200 μm.

**Figure S3** Principal components analysis and DEGs among four samples. The three biological replicates of each sample are clustered together in one ellipse using the principal components analysis **(A)** and the number of DEGs (Up and down) among four groups **(B)**.

**Figure S4** Heatmap of the DEGs in hormone pathways and the content of ZA in young spikes of WT and *ptsd1*. Heatmap of the DEGs involved in ethylene (ET) **(A)**, cytokinin (CK) **(B)** between WT-YS and *ptsd1*-YS. The content of Zeatin (ZA) **(C)** is decreased significantly in mutant *ptsd1.* The detail was listed in **Table S8**. The asterisks represent the significant difference between WT and *ptsd1* (**: *p* ≤ 0.01).

**Figure S5** Hierarchical cluster analyses of the differentially expressed TF gene families. Heatmap represents overall trend of the differential expression profiles of the 31 different TF families. The x-axis indicates the number of DEGs in different TF families. The y-axis represents the TF families. The detail was listed in **Table S9**.

**Figure S6** Cluster and expression pattern of the DEGs in MADS-box family. The following four parts are shown from left to right. Protein sequence maximum likelihood cluster tree **(Ⅰ)**: the amino acid sequences of the DEGs were listed in **Table S11**. Gene expression pattern **(Ⅱ)**: the gene expression levels were determined by RNA-seq data. Gene name **(Ⅲ)**: the homologous genes of the DEGs in NCBI. Gene family **(Ⅳ)**: the MADS-box subfamilies of the DEGs. The detail was listed in **Table S10**.

**Figure S7** The DEGs in gene families of receptor-like kinases, protein kinases, phosphatases and calcium signaling between WT-YS and *ptsd1*-YS. The x-axis indicates the number of DEGs. The y-axis represents receptor-like kinase, kinase, phosphatase and calcium-binding protein families. The detail was listed in **Tables S12,13**.

**Figure S8** ROS signaling in *ptsd1.* **(A)** Heatmap of ROS response-related gene expression patterns in different samples. The colour bar indicates expression levels from low (green) to high (red). **(B)** ROS are signals and modulators of plant defense responses and PCD. The up-regulated expression genes in *ptsd1* were marked in red. The detail was listed in **Table S14**.

**Figure S9** Heatmap of DEG families involved in macromolecule degradation **(A)**, nutrient transport **(B)**, N recycling **(C)** and secondary metabolites biosynthesis **(D)** between WT-YS and *ptsd1*-YS. The detail was listed in **Tables S17–20**.

**Figure S10** The distribution of starch (red) and protein (yellow) in wheat spike apexes of WT **(A)** and mutant *ptsd1* **(B)** at initiation of second awn stage. Scale bar = 200 μm.

**Figure S11** Heatmap of the DEGs encoding GST and glutathione-conjugate transporter. The annotation information was listed in **Table S18**.

**Figure S12** *K*-means clusters of the differentially expressed miRNAs and the qRT-PCR verifying. **(A)** K-means clusters of the differentially expressed miRNAs. The y-axis shows normalized fold-changes of the miRNA expressions and the x-axis indicates each sample, the average values of the three biological replicates are represented. **(B)** The qRT-PCR results of the miRNAs. The x-axis indicates samples. The left y-axis indicates the relative expression levels determined by qRT-PCR and the expression values are adjusted by setting the minimum expression to be 1 for each miRNA. The right y-axis indicates the TPM.

**Figure S13** DE miRNAs and their targets involved in senescence related signal responses and metabolisms in mutant *ptsd1*. The detailed information of the DE miRNAs and their targets is listed in **Table S24**. The miRNAs and targets coloured in red are highly expressed in young spikes of *ptsd1*, miRNAs and targets coloured in green are lowly expressed in young spikes of *ptsd1.*
